# Head and pelvic vertical displacement in dogs with induced swinging limb lameness: an experimental study

**DOI:** 10.1186/s13028-018-0435-z

**Published:** 2018-12-29

**Authors:** Anna Bergh, Constanza Bernardita Gómez Álvarez, Marie Rhodin, Pia Gustås

**Affiliations:** 1 Faculty of Veterinary Medicine and Animal Husbandry, Swedish University of Agricultural Sciences, 750 07 Uppsala, Sweden; 2 School of Veterinary Medicine, Faculty of Health & Medical Sciences, University of Surrey, Guildford, GU2 7AL UK

**Keywords:** Canine, Gait analysis, Kinematics, Lameness examination

## Abstract

**Background:**

Swinging limb lameness is defined as a motion disturbance ascribed to a limb in swing phase. Little is known about its biomechanics in dogs, particularly about the body motions that accompany it, such as vertical head and pelvic motion asymmetry. The aim of this study was to describe the changes in vertical head and pelvic motion asymmetry in dogs with induced swinging limb motion disturbance, mimicking a swinging limb lameness. Fore- and hind-limb lameness was induced in ten sound dogs by placing a weight (200 g) proximal to the carpus or tarsus, respectively. Marker-based motion capture by eight infrared light emitting video cameras recorded the dogs when trotting on a treadmill. Body symmetry parameters were calculated, including differences between the two highest positions of the head (HDmax) and pelvis (PDmax) and between the two lowest positions of the head (HDmin) and pelvis (PDmin), with a value of zero indicating perfect symmetry.

**Results:**

Induction of swinging forelimb lameness showed significant changes in HDmax (median and range: sound 1.3 mm [− 4.7 to 3.1], in the left side − 28.5 mm [− 61.2 to − 17.9] and in the right side 20.1 mm [− 4.4 to 47.5]) and, induction of swinging hind limb lameness showed significant changes in PDmax (sound 2.7 mm [− 7.4 to 7.2], in the left side − 10.9 mm [− 22.4 to 0.5] and in the right side 8.6 mm [− 3 to 30]), as well as an increased hip movement asymmetry (sound 1.6 mm [− 8.6 to 19.9], in the left side − 18.1 mm [− 36.7 to 5.4] and in the right side 15 mm [− 20.7 to 32.1]) (P < 0.05).

**Conclusions:**

Induced swinging fore- and hind limb lameness resulted in significant increased asymmetry of the maximal vertical displacement movement of the head and pelvis, due to decreased lifting of the head in forelimb lameness and of the pelvis in hind limb lameness. The results suggest that asymmetry of the maximal vertical displacement of the head and pelvis (i.e. lifting) is a key lameness sign to evaluate during examination of swinging limb lameness.

## Background

Normal canine gaits, such as walk and trot, are characterized by symmetry in weight bearing and body and limbs’ motion, comparing the dog’s left and right side. Consequently, a systematically recurring divergence from motion symmetry is a potential sign of functional impairment, pain or mechanical disturbance—a lameness. Lameness can be present as primarily a supporting limb lameness (weight bearing lameness), with adjustments of the body center of mass registered during the stance phase of the stride cycle. It can also be present as a swinging limb lameness, a motion disturbance ascribed to a limb during swing phase, with characteristic motion patterns depending on the location of the injury. In naturally occurring conditions, studies have shown significant changes in the swinging pattern of limbs in dogs with infraspinatus muscle contractures causing the foot to swing laterally during protraction or in gracilis muscle contracture causing hyperflexion and outward rotation of the tarsus with inward rotation of the foot [1, 2], as well as in the paw velocity and stifle angular velocity in dogs with rupture of the cranial cruciate ligament (CCL) [3]. However, the studies did not investigate the symmetry of vertical head and pelvic movement. Other causes of swinging limb lameness are specific joint disorders such as ankylosis or those causing elbow, shoulder, hip or stifle pain; where also specific angular motion changes occur on the affected limb, for example, abnormal joint flexion–extension, rotation, abduction–adduction and protraction-retraction [3–14]. However, these changes in limbs movement are more likely to be presented combined with supporting limb lameness features because the affected joint might be painful or uncomfortable during both stance phase and swing phase causing also changes in limb loading [15–19]. To the authors’ knowledge, the head and pelvic motion during swinging limb lameness has not been described. Kinetic studies of naturally occurring orthopedic conditions in proximal joints have shown that limb loadings are transferred from a painful limb to the non-painful limb, which is reversed with successful surgical treatment, particularly peak breaking force in cruciate ligament injuries and vertical forces when a proximal joint is affected [20–27]. Similarly, kinematic studies have shown differences in stride length and duration, joint velocity and joint range of motion between sound dogs and dogs with different types of clinical conditions of the elbow, hip and stifle [3, 6, 7, 9–11, 13, 26].

Visual lameness examination is used to localize, characterize and grade the lameness by observing compensatory movement patterns during the stance and swing phase. Besides of including the assessment of the vertical motion of the head, as well as of the pelvis and the hip; the so called “head nod” and “hip hike”. This is regarded as fundamental variables in the visual lameness examination [2, 28]. It has been shown that a supporting forelimb lameness results in an asymmetry of the vertical head motion, with the lowest head position observed during the stance phase of the sound forelimb [4, 29–31] and that a supporting hind limb lameness results in an asymmetry of the pelvic motion, with the lowest pelvic position observed during the initial and mid-stance phase of the sound hind limb [30, 31]. Further, supporting hind limb lameness increases the overall vertical hip motion on the affected side during the stance phase of the sound limb, showing a hip movement asymmetry (“hip hike”) on the affected side [31]. Furthermore, a primary supporting fore- or hind limb lameness may result in additional movements of the hip and head, respectively, referred to as a “false” or compensatory lameness [30, 31]. Despite the prevalent use of the “head nod” and “hip hike” in the visual lameness investigation of dogs, little is known on how the motion alters during different types of lameness. Quantification of head and pelvic motion in dogs during lameness (induced or clinical) has so far only been reported in a few studies [14, 30, 31]. The first [14] showed a correlation between a greater mean total pelvic vertical motion and a higher peak vertical force, registered during the stance phase of the sound hind limb, in dogs with subtle clinical stifle problems. Compared to supporting limb lameness, even less is known about the body compensatory movement patterns during swinging limb lameness. A broadened understanding of compensatory movements in this type of lameness in dogs could help improving the sensitivity of lameness detection during visual lameness examination.

Therefore, the aim of the current study was to quantify the vertical motion symmetry of the head, pelvis and hip in dogs before and after an induced limb motion disturbance resembling swinging limb lameness. The hypothesis was that induced swinging fore- and hind-limb lameness in trotting dogs will alter vertical movement symmetry of head, pelvic and hip compared to sound registrations.

## Methods

The study design was approved by the local Ethical Committee on Animal Experiments at of the University of Agricultural Sciences, Sweden (C283/12, 1 February 2013) and performed with informed consent of the owners.

### Dogs

Ten clinically sound dogs were used in the present study (five Labrador retrievers, one each of Flat coated retriever, Australian shepherd, Dalmatian, Lagotto Romagnolo and Irish terrier; two neutered males, one intact male and seven intact females; age: [mean ± standard deviation, SD] 5.1 ± 1.2 years; body weight 23.4 ± 6.0 kg; height at the withers: 53.0 ± 5.5 cm). Dogs were assessed as clinically sound based on an orthopedic examination including a visual gait assessment at walk and trot. Eight dogs had radiographic screening free of hip and elbow dysplasia according to the Federation Cynologique Internationale screening protocol (Grade A or B), the remaining dogs had not undergone screening.

### Motion analysis system

A motion capture system consisting of eight Oqus 121 infrared light emitting video cameras (Qualisys AB, Gothenburg, Sweden), set at 240 Hz, was used to capture the movement of the dogs while trotting on a treadmill (Rodby Innovation AB, Vänge, Sweden) at their own comfortable speed (mean ± SD 1.9 ± 0.1 m/s), which was registered and repeated for each dog. Five clusters of reflective markers, with three markers of 7 mm in diameter in each cluster, were attached to five inertial sensors (synchronized for a parallel study on lameness detection) by double-adhesive tape, placed on the frontal bone (the midline top of the head), the dorsal aspect of the metacarpus of the right forelimb, both greater trochanters (hip) and the midline pelvis (median sacral crest). Data was collected before and after transient lameness induction described below.

### Study protocol

**Fig. 1:**
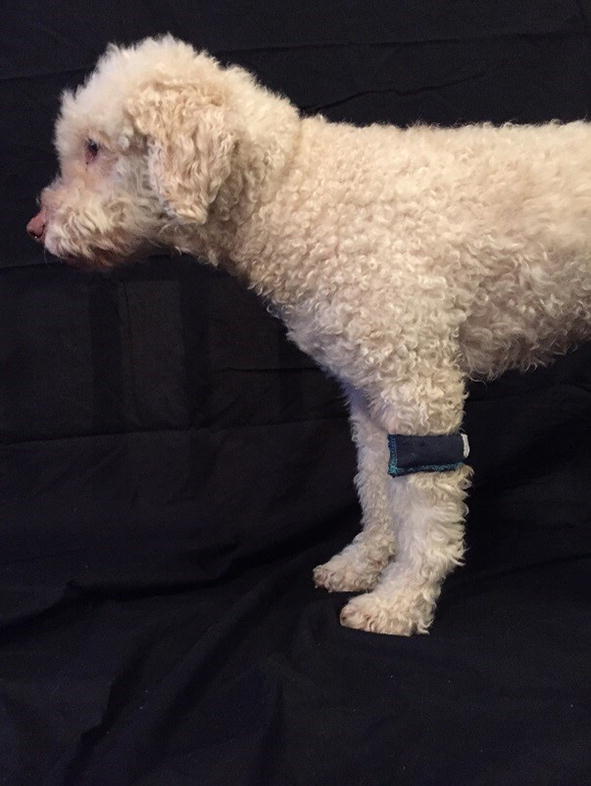
Weight positioned proximal to the carpus

**Fig. 2:**
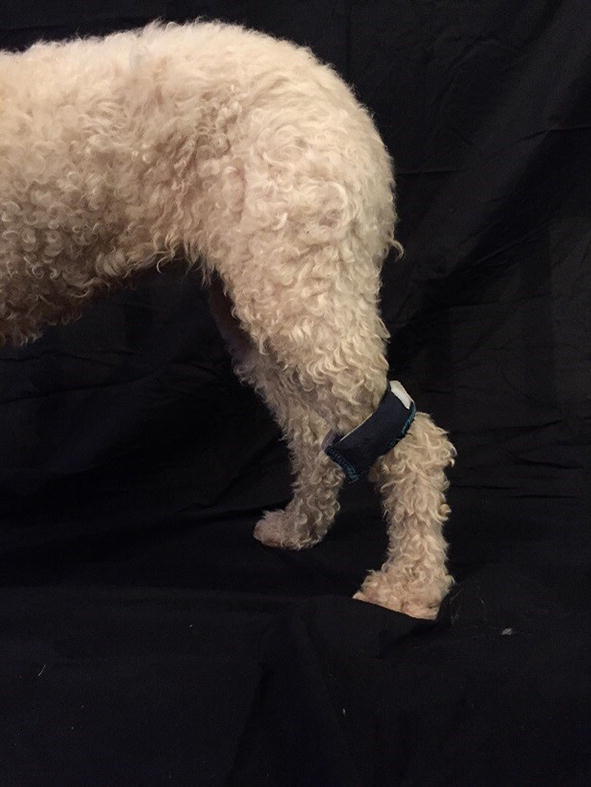
Weight positioned proximal to the hock

Before start of the experiment, the dogs were accustomed to treadmill trotting during four sessions as described elsewhere [32]. Before the first recording each dog had a warm-up period of approximately 10 min at walk and trot. The speed of the treadmill was individually set based on what was visually assessed as comfortable to the dog and measurement was started when the dog was trotting at a steady gait. Recordings were done during 20 s periods and the experiment was video recorded from a lateral and caudal aspect, in order to validate registrations for data analysis. A registration was considered valid when the dog trotted at an even pace and with the head centered looking straight forward. Each session started with an initial standing square position recording. Thereafter, the dogs were recorded while trotting on the treadmill (“sound registration”). Two types of lameness (supporting and swinging limb lameness) were randomly induced in all fore- and hind limbs, one limb at a time (results from our parallel study regarding the supporting limb lameness is presented in Gómez Álvarez et al. [31]). The dogs were randomly assigned to one of 10 different orders of induction by drawing a number from a container. A pre-set degree of swinging limb lameness (moderately lame, distinctly visible at the trot, 2 degrees on a scale of 0–5) was induced by placing the custom made weight (200 g), proximal to the carpus and to the tarsus, respectively (Figs. 1, 2). Between each registrations of induced lameness, sound control measurements were performed to ensure return to soundness. The return to soundness was ensured by visual examination by two veterinarians. The dogs had a “wash-out” consisting of a short rest followed by a short warm-up before the control sound registration. In total, the experimental session consisted of sixteen registration periods, where eight belonged to the swinging limb lameness data.

### Data analysis

**Fig. 3:**
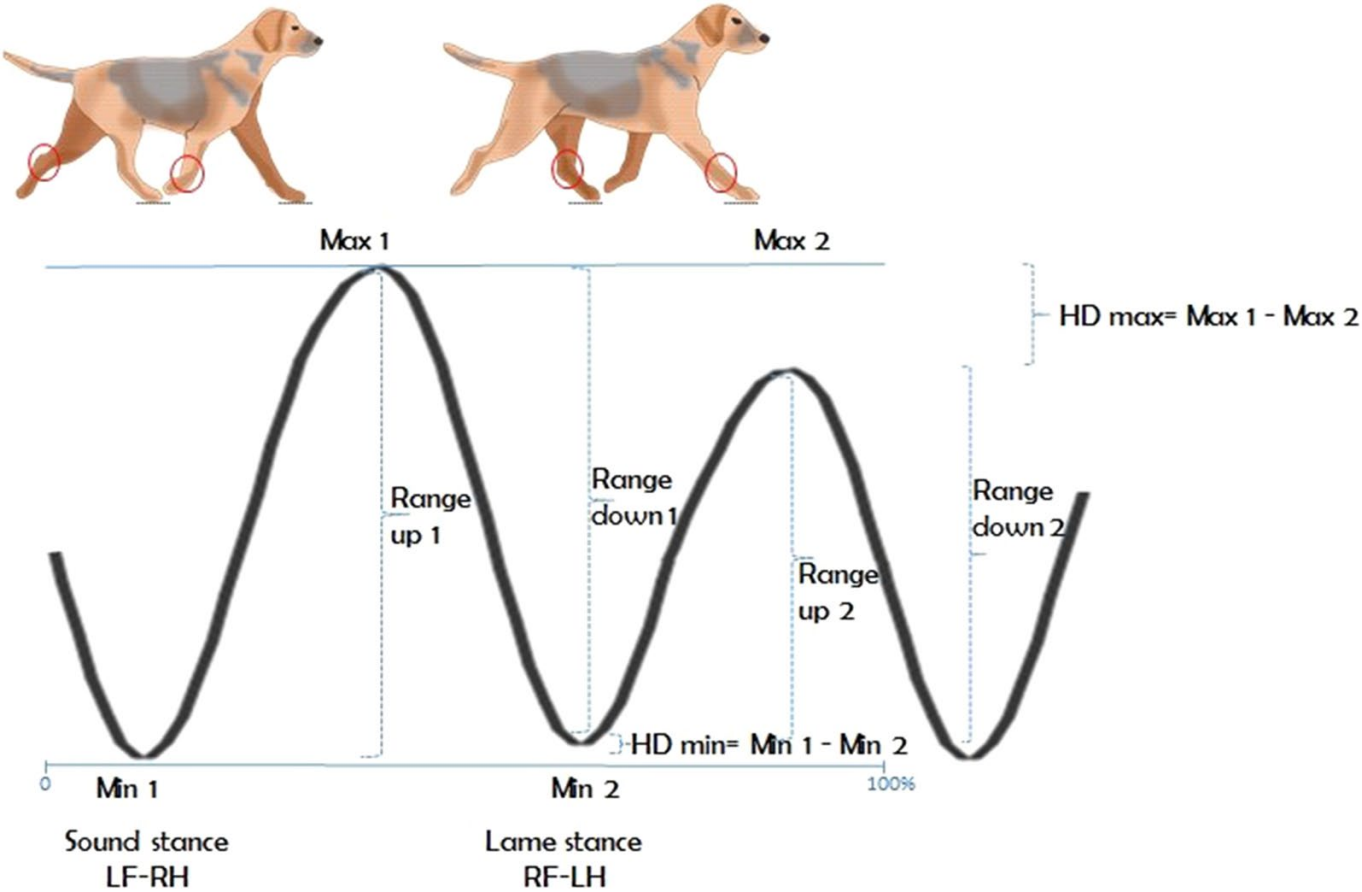
Schematic description of the vertical motion of the head during a stride cycle, where pelvis shows a similar curve where min1 represents the lowest position during left hind stance. For both head and pelvis the following formulas were used: Range down differences = (Range down 1 − Range down 2), Range up differences = (Range up 1 − Range up 2), SI up = (Range up 1 − Range up 2)/Max Range up; SI down = (Range down 1 − Range down 2)/Max Range down

The kinematic data was processed and three-dimensionally reconstructed with motion analysis software (Qualisys Track Manager, Qualisys AB, Gothenburg, Sweden). A custom made script in Matlab (The MathWorks, Inc, MA, USA) was used for further analyses as described in [31]. The vertical displacement of the head, pelvic and greater trochanters markers was reconstructed for all strides per trial. The maximal protraction of the left metacarpal marker was used to split the data into strides. The values of the two highest and lowest head positions and the two highest and lowest pelvic positions were extracted. The differences, in mm, between the two highest vertical displacements of the head (HDmax) and pelvis (PDmax) and between the two lowest displacements of the head (HDmin) and pelvis (PDmin) were computed per stride. For HDmax/PDmax, a positive value indicates a higher position after left limb stance, compared to after right limb stance. Further, for HDmin/PDmin, a positive value indicates a lower position during left limb stance, compared to right limb stance. The difference, in mm, between the two upward motion ranges of the head (range up HD) and pelvis (range up PD) and the two downward motion ranges (range down HD and PD) were also calculated (Fig. 3). Further, symmetry indices of the head and pelvic upward movement ranges (SIup) and downward movement ranges (SIdown) were calculated by using the following formulas modified from Starke et al. [33].

$${\text{SI}}\;{\text{up}} = \left( {{\text{Range}}\;{\text{up}}\;1{-}{\text{Range}}\;{\text{up}}\;2} \right)/{\text{Max}}\;{\text{Range}}\;{\text{up}};$$

$${\text{SI}}\;{\text{down}} = \left( {{\text{Range}}\;{\text{down}}\;1{-}{\text{Range}}\;{\text{down}}\;2} \right)/{\text{Max}}\;{\text{Range}}\;{\text{down}} .$$

A value of zero indicates perfect symmetry, whilst for range up/SI up a negative value indicates a smaller upward range of motion after left limb stance and a positive value indicates a smaller upward range of motion after right limb stance. For range down/SI down a negative value indicates a larger downward range of motion before left limb stance and a positive value indicates a larger downward range of motion before right limb stance. Max range up/Max range down are the largest range values (i.e. range up or down 1 and range up or down 2) [33].

The difference, in mm, between the vertical displacements of the left and right greater trochanters during swing phase (hip movement asymmetry or “hip hike difference”) were calculated. Negative values indicate a greater vertical movement of the left trochanter, compared to the movement of the right trochanter. Stride duration was also calculated.

### Statistics

The data is presented as median and range, unless other is stated. Medians and ranges for all variables before and after induction of lameness were calculated. Normality was tested with D’Agostino & Pearson normality test. Data were analyzed using Friedman’s test and Dunn’s post hoc test in GraphPad Prism (GraphPad Company, San Diego, CA, USA). Significance was set at P < 0.05.

## Results

Normality test showed the data was not normally distributed, therefore a nonparametric test was used for statistical analysis. Friedman-test was used to identify significant differences between induced lameness in different limbs between dogs and Dunn’s post hoc test was used to confirm where the differences occurred between groups.

### Data collection

Kinematic data from five registrations periods (one sound and one for each of the induced swinging limb lameness) was analyzed for each of the ten dogs, a total of nine trials and an average of mean ± SD of 39.2 ± 1.1 strides per trial and dog (for all kinematic data see Table 1). There were no significant differences in stride duration between trials before and after lameness induction.

**Table 1:** Median (range) values for symmetry parameters in 10 dogs with induced swinging limb disturbance (mimicking fore- and hind limb lameness) at a mean ± standard deviation trotting speed of 1.9 ± 0.1 m/s

Parameter	Sound	Forelimb lameness		Hind limb lameness	
		Left forelimb	Right forelimb	Left hind limb	Right hind limb
HDmin (mm)	4.6 (− 5.6 to 11)	− 3.2 (− 19.2 to 22.8)	2.6 (− 12.5 to 18.4)	− 4.8 (− 10.8 to 6.3)	0.05 (− 10.1 to 19.8)
HDmax (mm)	1.3 (− 4.7 to 3.1)	− *28.5* (− *61.2* to − *17.9*)*	*20.1* (− *4.4* to *47.5*)*	− 5.1 (− 11.9 to 6.9)	2.6 (− 1.6 to 21.0)
PDmin (mm)	1.0 (− 2.2 to 4.1)	− 1.5 (− 12.6 to 10.6)	2.9 (− 0.7 to 13.5)	0.7 (− 8.0 to 12.0)	0.4 (− 8.5 to 11.4)
PDmax (mm)	2.7 (− 7.4 to 7.2)	− 0.5 (− 11.1 to 10.5)	3.8 (− 16.9 to 17.8)	− *10.9* (− *22.4* to *0.5*)*	*8.6* (− *3.0* to *30.0*)*
Range up HD (mm)	4.4 (− 12.2 to 11.8)	− *35.0* (− *65.0* to − *26.6*)*	*27.3* (− *4.0* to *56.2*)*	− 6.0 (− 22.3 to 9.4)	6.4 (− 9.1 to 37.0)
Range down HD (mm)	− 2.9 (− 9.8 to 4.2)	− 30.6 (1.2 to − 65.8)	16.8 (− 8.2 to 50.4)	1.0 (− 14.7 to 16.9)	4.3 (− 12.2 to 8.6)
Range up PD (mm)	4.4 (− 7.6 to 10)	− 2.0 (− 17.2 to 16.8)	8.7 (− 17.0 to 25.9)	− *9.8* (− *21.2* to *6.1*)*	*9.9* (− *8.5* to *27.9*)*
Range down PD (mm)	1.9 (− 6.4 to 5.7)	5.3 (− 18.3 to 14.7)	− 0.8 (− 16.6 to 18.5)	− *8.6* (− *34.3* to *1.4*)*	6.6 (− *2.9* to *44.5*)*
SIup H	0.2 (− 0.5 to 0.4)	− *0.8* (− *1.1* to − *0.6*)*	*0.6* (− *0.2* to *1.0*)*	− 0.3 (− 0.6 to 0.4)	0.3 (− 0.4 to 0.8)
SIdown H	− 0.1 (− 0.3 to 0.2)	− *0.7* (− *1.1* to *0.1*)*	*0.5* (− *0.4* to *1.0*)*	0.0 (− 0.5 to 0.6)	0.1 (− 0.3 to 0.5)
SIup P	0.1 (− 0.2 to 0.4)	0.0 (− 0.5 to 0.4)	0.2 (− 0.4 to 0.7)	− *0.2* (− *0.5* to *0.2*)*	*0.3* (− *0.2* to *0.6*)*
SIdown P	0.1 (− 0.2 to 0.1)	0.1 (− 0.6 to 0.4)	0.0 (− 0.4 to 0.5)	− *0.2* (− *0.6* to *0.0*)*	*0.2* (− *0.1* to *0.6*)*
Hip movement asymmetry (mm) during swing phase	1.6 (− 8.6 to 19.9)	− 4.3 (− 27.6 to 8.2)	4.9 (− 21.4 to 24.5)	− *18.1* (− *36.7* to *5.4*)*	*15* (− *20.7* to *32.1*)*
Stride duration (ms)	514 (426 to 568)	529 (477 to 586)	534 (446 to 565)	506 (434 to 562)	512 (446 to 551)

Difference between the two highest displacements of the head (HDmax) and mid-pelvis (PDmax); difference between the two lowest displacements of the head (HDmin) and mid-pelvis (PDmin); differences between the two upward movements of the head and mid-pelvis (range up HD, range up PD); differences between the two downward movements of the head and mid-pelvis (range down HD, range down PD); a value of zero indicates perfect symmetry, whilst negative and positive values indicate a left or right limb lameness, respectively; symmetry indices of the head and mid-pelvis upwards movement (SI up H, SI up P) and downwards movements (SI down H, SI down P), with a value of zero indicating perfect symmetry; difference between the left and right hip vertical displacement (Hip asymmetry during swing phase), where negative and positive values indicate a left or right limb lameness, respectively. Data are expressed in mm unless specified otherwise. Significant differences (P < 0.05) compared with sound trials are written in italics and indicated with asterisks (*)

### Differences in highest and lowest positions

Minor, non-significant head and pelvic motion asymmetries, however not clinically observed, were detected during the sound trials.

Results are presented as medians and ranges. There was a significantly increased absolute value of HDmax during induced swinging left forelimb lameness (− 28.5 mm [− 61.2 to − 17.9], P = 0.026) and in right forelimb lameness (20.1 mm [− 4.4 to 47.5], P = 0.021) compared to the HDmax before induction (1.3 mm [− 4.7 to 3.1]). Similarly, there was a significantly increased absolute value of PDmax during induced swinging left hind limb lameness (− 10.9 mm [− 22.4 to 0.5], *P* = 0.004) and during induced right hind limb lameness (8.6 mm [− 3 to 30], P = 0.004) compared to the PDmax before induction (2.7 mm [− 7.4 to 7.2]). There were no significant changes in HDmin or PDmin. These results indicate that swinging forelimb lameness induction resulted in a reduced maximum position of the head after the lame forelimb push-off and during the swinging of the affected forelimb, and that swinging hind limb lameness induction resulted in a reduced maximum position of the pelvis after push-off, and swinging of the affected hind limb.

### Range up and down, and symmetry indices

There were significantly increased absolute values of range up HD during induced swinging left forelimb lameness (− 35.0 mm [− 65.0 to − 26.6], P = 0.002) and during right forelimb lameness (27.3 mm [− 4.0 to 56.2], P = 0.015) compared to before induction (4.4 mm [− 12.2 to 11.8]). Similarly, there were significantly increased absolute values of range up PD during induced swinging left hind limb lameness (− 9.8 mm [− 21.2 to 6.1], P = 0.011) and during right limb lameness (9.9 mm [− 8.5 to 27.9], P = 0.013) compared to before induction (4.4 mm [− 7.6 to 10]). There was also a significantly increased absolute value of range down PD during induced left swinging hind limb lameness (− 8.6 mm [− 34.3 to 1.4], P = 0.026) and during right limb lameness (6.6 mm [− 2.9 to 44.5], P = 0.014) compared to before induction (1.9 mm [− 6.4 to 5.7]). There were no significant changes in range down HD. Further, the symmetry indices significantly changed during induced left swinging forelimb lameness for SIup H (− 0.8 [− 1.1 to − 0.6], P = 0.024) and for right forelimb lameness (0.6 [− 0.2 to 1.0], P = 0.03) and SIdown H for left forelimb lameness (-0.7 [− 1.1 to 0.1], P = 0.011) and for right forelimb lameness (0.5 [− 0.4 to 1.0], P = 0.022) compared to the sound registrations (SIup H: 0.2 [− 0.5 to 0.4] and SIdown H: − 0.1 [− 0.3 to 0.2]). Significant changes were also seen during induced left hind limb lameness for SIup P (− 0.2 [− 0.5 to 0.2], P = 0.035) and during right hind limb lameness (0.3 [− 0.2 to 0.6], P = 0.035), compared to sound (0.1 [− 0.2 to 0.4]. SIdown P for left hind limb lameness (− 0.2 [− 0.6 to 0.0], P = 0.108) and for right hind limb lameness (0.2 [− 0.1 to 0.6], P = 0.23) compared to the sound registrations (SIdown P: 0.1 [− 0.2 to 0.1]).

### Hip movement asymmetry (“hip hike differences”)

During the swing phase of induced hind limb lameness, there was a significantly increased absolute value of hip movement asymmetry in left limb lameness (− 18.1 mm [− 36.7 to 5.4], P = 0.021) and in right limb lameness (15 mm [− 20.7 to 32.1], P = 0.044), compared to the sound registrations (1.6 mm [− 8.6 to 19.9]), with an increased displacement of the greater trochanters during the lame limb swing phase.

## Discussion

To our knowledge, the present study is the first to investigate the motion pattern of the body during induced limb motion disturbance, mimicking a swinging limb lameness. The results show a significant increase in the difference between the two highest positions of the head (HDmax) in swinging forelimb lameness and between the two highest positions of the pelvis (PDmax) in hind limb lameness, which means between the two head or pelvic lifting during one stride cycle, respectively. This, together with the results from our previous study showing a significant increase in the HDmin and PDmin during supporting limb lameness [31], confirms that changes in the head, pelvic and hip vertical movement can serve as good indicators for both supporting and swinging fore- and hind limb lameness, respectively, and that changes in head or pelvis lifting seem to be characteristic of swinging limb lameness, while changes in head or pelvis lowering seem to be characteristic of supporting limb lameness, at least in an experimental situation. Little is known about how lameness originated proximally in the limb differ from those originated more distally, therefore the results of the present study are contributing to the better understanding of the differences between the two type of lameness. These findings are also supported by our parallel study were the lameness of the same dogs was simultaneously detected with an inertial measurement system [30], where significant changes in HDmax during induced swinging hind limb lameness were detected, mimicking the head motion asymmetry normally observed during forelimb lameness. This was not significant in the present study. The most likely explanation for this is the different sensitivity of the tests used for statistical analysis. The results of our study agree with the increased vertical displacement of the pelvis during the sound stance in clinical hind limb lameness reported by Hicks and Millis in dogs with CCL rupture [14]. However, it does not agree with the head dropping and pelvis dropping during the sound stance described in shoulder osteochondrosis and CCL rupture, respectively, by Leach et al. [4]. These differences are probably due to the different degree and nature of our induced lameness disturbance where no pain is produced, compared to the biomechanics of clinical cases, where pain or mechanical limitations decrease the ability to swing the limb in its normal arc and to absorb the weight during stance phase [4].

The general aim of the present study was to investigate if swinging limb lameness affects the movement of the head and pelvis, in order to present more detailed data on what movement patterns to look for in a visual lameness assessment and to investigate the magnitude of these motion patterns aiming to further investigate parameters used in lameness quantification using motion sensors. Since there is limited data on the movement of head and pelvis during swinging limb lameness, the study was performed on sound dogs with induced lameness. The use of sound dogs with induced lameness enabled a reduced variability in lameness compared to the use of patients. The ability to induce a temporary lameness offered an important and reliable method to study the motion pattern during this specific, well-defined lameness in a controlled manner that minimizes individual variations. This lameness model aimed to mimic the motion patterns occurring during soft tissue pathologies causing swinging lameness. Research on the mechanical adaptations of naturally occurring lameness of specific pathologies affecting both, supporting and swinging phase of the stride cycle are warranted. The results from our study build a base for future research, looking at naturally occurring lameness. In the future, studies on patients with detailed description of each patient’s movement pattern will further help clinicians in their daily work evaluating canine lameness.

Specific limb motion changes occur during swinging limb lameness, such as those shown in kinematic studies in dogs with stifle osteoarthritis, where dogs present an earlier flexion of the hip during swing phase of the affected limb, as well as a faster extension of the hip during early stance and an increased stifle flexion during the end of the stance phase- influencing the braking potential [24]. These dogs are suggested to use their hip more than usual for braking and propelling-changing the movement of the pelvis and hip [7], which is in agreement with the significant change in hip movement asymmetry observed in the present study. Hicks and Millis [14] have shown greater pelvic vertical motion during the stance phase of the sound hind limb in dogs with subtle clinical lameness due to CCL injury, similarly to our study. They have also shown a significant change in the thoracolumbar lateral angular motion toward the affected hind limb presenting reduced weight bearing, which indicates that not only vertical motion asymmetry of the body is a key lameness indicator but also medio-lateral motion [14].

There are some limitations to the present study. Although the experimental setting does not necessary reflect a clinical situation, we choose to induce lameness instead of using patients. A controlled experimental environment helps to reduce the variability in the results, a variability that may be introduced by differences in speed, body size and lameness etiology in a clinical material. The mimicking of a swinging limb lameness achieved by adding a weight to the limbs in this study is a non-painful lameness induction which does not equate to a painful joint. Due to ethical reasons and the high variability of the pain-induced model; a painful, reversible swinging lameness induction was not considered. It is possible that pain-induced lameness affects compensatory mechanisms additionally to those that are weight-related. However, it has been shown that the addition of mass to the canine wrist affects the forelimb protractor and retractor muscle activity during swing phase [34], and studies in horses report changes in the swinging phase when adding weights to the limbs, thus indicating that a weight affects the mechanical events occurring during swing phase, therefore it mimics a swinging limb lameness [35, 36]. The amount of weight was selected based on practical experience on using weights for rehabilitation purposes. The dogs responded to the weight in a similar way, assessed by visual examination by a person looking at the dog from the side and from the front. The grade of lameness was also confirmed after the experiment- from a video recording. Based on visual assessment the dogs did not show an obvious increased lateral movement pattern, as dogs with natural m. infraspinatus contracture often does. It is likely that dogs with that pathology presenting an exaggerated lateral movement pattern would have an enhanced effect on the vertical head and pelvic movement as well.

Limbs motion was not analyzed in our study, which would have given a wider picture of the adaptations that take place in swing limb lameness. However, most of the objective measurement technology currently used in equine lameness assessment is based on head and pelvic vertical motion asymmetry. Thus, this study contributes to improve the progresses into objective lameness detection in dogs.

Despite the fact that no lameness was visually detected during the sound registrations between lameness inductions, some mild asymmetries were registered by the motion analysis system. These might have lessened the difference between lame and sound registrations, and may be the cause of some inconsistencies in the sign (i.e. plus or minus) of the values reported, indicating left or right lameness when induction was performed in the opposite side. All dogs had only minor asymmetries recorded by the objective measures during the studies sound registrations. Although a low level of asymmetrical gait is not unique to sound populations of dogs [37], thresholds for normal variation asymmetry versus asymmetry due to lameness have not been identified in dogs yet. Further investigation is needed to establish such thresholds, particularly to further develop the use of automated lameness detectors in canine.

Finally, the study was performed with dogs trotting on a treadmill at their own comfortable speed, which varied between individuals due to their size, however, the speed range of the dogs were in accordance with other studies and did not show differences between trials for each individual [6, 38, 39]. It is known that the stride frequency increases and stride length decreases when trotting on a treadmill compared to trotting over ground at the same velocity [40].

The results of our study suggest that asymmetry of the maximal displacement of head and pelvis (i.e. lifting) is the key lameness sign to evaluate during examination of swinging limb lameness. Conversely, our previous study on supporting limb lameness have shown that during forelimb lameness, the minimal displacement of head and pelvis (i.e. head nod and hip drop) is the key lameness sign to evaluate [31]. However, it would be difficult for a clinician to recognize asymmetries in lifting head or pelvis vs asymmetries in lowering the head or pelvis if the lameness is mild and/or intermittent. These findings help to better understand and differentiate the mechanisms of swinging and supporting limb lameness, and contribute to the knowledge needed to further develop objective lameness detection (i.e. inertial motion measuring units) for quantification of motion asymmetries in canines during the lame swing phase. Although locomotion asymmetry does not equal to lameness, it helps to the decision-making process during visual lameness examination using both measures of asymmetry and clinical findings.

## Conclusions

In sound dogs, induction of a swinging limb lameness causes motion asymmetry of the head, pelvis and hip, compared to sound registrations, decreasing the lifting of the head or pelvis. Based on our results, there are indications that a primary swinging lameness results in a reduced highest position of the head after the lame forelimb push-off and during the swinging of the affected forelimb, and a primary hind limb lameness results in a reduced highest position of the pelvis after push-off, and swinging of the affected hind limb and; an increased hip movement asymmetry, with an increased vertical motion of the weighed side during its swing phase. The results suggest that asymmetry of the maximal vertical displacement of head and pelvis (i.e. lifting) is the key lameness sign to evaluate during swinging limb lameness. Further studies are needed to compare these results to the pattern of movement in different types of clinical lameness, as well as during over ground locomotion.

## References

[1] Eward C, Gillette R (2003). Eward W Effects of unilaterally restricted carpal range of motion on kinematic gait analysis of the dog. Vet Comp Orthop Traumatol.

[2] Houlton J, Houlton JEF, Cook JL, Innes JF, Langley-Hobbs SJ (2006). An approach to the lame dog or cat. BSAVA manual of canine and feline musculoskeletal disorders.

[3] Sanchez-Bustinduy M, de Medeiros MA, Radke H, Langley-Hobbs S, McKinley T, Jeffery N (2010). Comparison of kinematic variables in defining lameness caused by naturally occurring rupture of the cranial cruciate ligament in dogs. Vet Surg.

[4] Leach D, Sumner-Smith G, Dagg AI (1977). Diagnosis of lameness in dogs: a preliminary study. Can Vet J.

[5] Korvick DL, Pijanowski GJ, Schaeffer DJ (1994). Three-dimensional kinematics of the intact and cranial cruciate ligament-deficient stifle of dogs. J Biomech.

[6] Bennet RL, De Camp CE, Flo G, Hauptman JG, Stajich M (1996). Kinematic gait analysis in dogs with hip dysplasia. Am J Vet Res.

[7] DeChamp CE, Riggs CM, Olivier NB, Hauptman JG, Hottinger HA, Soutas-Little RW (1996). Kinematic evaluation of gait in dogs with cranial cruciate ligament rupture. Am J Vet Res.

[8] Tashman S, Anderst W, Kolowich P, Havstad S, Arnoczky A (2004). Kinematics of the ACL-deficient canine knee during gait: serial changes over two years. J Orthop Res.

[9] Bockstahler BA, Henninger W, Muller M, Mayrhofer E, Peham C, Podbregar I (2007). Influence of borderline hip dysplasia on joint kinematics of clinically sound Belgian Shepherd dogs. Am J Vet Res.

[10] Bockstahler BA, Vobornik A, Müller M, Peham C (2009). Compensatory load redistribution in naturally occurring osteoarthritis of the elbow joint and induced weight-bearing lameness of the forelimbs compared with clinically sound dogs. Vet J.

[11] Bockstahler BA, Prickler B, Lewy E, Holler PJ, Vobornik A, Peham C (2012). Hind limb kinematics during therapeutic exercises in dogs with osteoarthritis of the hip joints. Am J Vet Res.

[12] Drüen S, Böddeker J, Meyer-Lindenberg A, Fehr M, Nolte I, Wefstaedt P (2012). Computer-based gait analysis of dogs: evaluation of kinetic and kinematic parameters after cemented and cementless total hip replacement. Vet Comp Orthop Traumatol.

[13] Miqueleto NS, Rahal SC, Agostinho FS, Siqueira EG, Araújo FA, El-Warrak AO (2013). Kinematic analysis in healthy and hip-dysplastic German Sherpherd dogs. Vet J.

[14] Hicks DA, Millis DL (2014). Kinetic and kinematic evaluation of compensatory movements of the head, pelvis and thoracolumbar spine associated with asymmetric weight bearing of the pelvic limbs in trotting dogs. Vet Comp Orthop Traumatol.

[15] Rumph PF, Kincaid SA, Visco DM, Baird DK, Kammermann JR, West MS (1995). Redistribution of vertical ground reaction force in dogs with experimentally induced chronic hind limb lameness. Vet Surg.

[16] Abdelhadi J, Wefstaedt P, Nolte I, Schilling N (2012). Fore-aft ground force adaptations to induced forelimb lameness in walking and trotting dogs. PLoS ONE.

[17] Abdelhadi J, Wefstaedt P, Galindo-Zamora V, Anders A, Nolte I, Schilling N (2013). Load redistribution in walking and trotting beagles with induced forelimb lameness. Am J Vet Res.

[18] Fischer S, Anders A, Nolte I, Schilling N (2013). Adaptations in muscle activity to induced, short-term hindlimb lameness in trotting dogs. PLoS ONE.

[19] Fischer S, Anders A, Nolte I, Schilling N (2013). Compensatory load redistribution in walking and trotting dogs with hind limb lameness. Vet J.

[20] Budsberg SC, Chambers JN, Lue SL, Foutz TL, Reece L (1996). Prospective evaluation of ground reaction forces in dogs undergoing unilateral total hip replacement. Am J Vet Res.

[21] Theyse LFH, Hazewinkel HAW, Van Den Brom WE (2000). Force plate analyses before and after surgical treatment of unilateral fragmented coronoid process. Vet Comp Orthop Traumatol.

[22] Evans R, Horstman C, Conzemius M (2005). Accuracy and optimization of force platform gait analysis in Labradors with cranial cruciate disease evaluated at a walking gait. Vet Surg.

[23] Fanchon L, Grandjean D (2007). Accuracy of asymmetry indices of ground reaction forces for diagnosis of hind limb lameness in dogs. Am J Vet Res.

[24] Madore E, Huneault L, Moreau M, Dupuis J (2007). Comparison of trot kinetics between dogs with stifle or hip arthrosis. Vet Comp Orthop Traumatol.

[25] Voss K, Imhof J, Kaestner S, Montavon PM (2007). Force plate gait analysis at the walk and trot in dogs with low-grade hindlimb lameness. Vet Comp Orthop Traumatol.

[26] Ragetly CA, Griffon DJ, Mostafa AA, Thomas JE, Hsiao-Wecksler ET (2010). Inverse dynamics analysis of the pelvic limbs in Labrador retrievers with and without cranial cruciate ligament disease. Vet Surg.

[27] Oosterlinck M, Bosmans T, Gasthuys F, Polis I, van Ryssen B, Dewulf J, Pille F (2011). Accuracy of pressure plate kinetic asymmetry indices and their correlation with visual gait assessment scores in lame and nonlame dogs. Am J Vet Res.

[28] Renberg WC (2001). Evaluation of the lame patient. Vet Clin Small Anim.

[29] Nunamaker, Boulder, Newton SD, Nunamaker (1985). Normal and abnormal gait. Textbook of small animal orthopaedics.

[30] Rhodin M, Bergh A, Gustås P, Gómez Álvarez CB (2017). Inertial sensor-based system for lameness detection in trotting dogs with induced lameness. Vet J.

[31] Gómez Álvarez CB, Gustås P, Bergh A, Rhodin M (2017). Vertical head and pelvic movement symmetry at the trot in dogs with induced supporting limb lameness. Vet J.

[32] Gustås P, Pettersson K, Honkavaara S, Lagerstedt AS, Byström A (2013). Kinematic and temporospatial assessment of habituation of Labrador retrievers to treadmill trotting. Vet J.

[33] Starke SD, Willems E, May SA, Pfau T (2012). Vertical head and trunk movement adaptations of sound horses trotting in a circle on a hard surface. Vet J.

[34] Carrier DR, Deban SM, Fischbein T (2008). Locomotor function of forelimb protractor and retractor muscles of dogs: evidence of strut-like behavior at the shoulder. J Exp Biol.

[35] Wickler SJ, Hoyt DF, Clayton HM, Mullineaux DR, Cogger EA, Sandoval E (2004). Energetic and kinematic consequences of weighting the distal limb. Equine Vet J.

[36] Clayton HM, Lavagnino M, Kaiser LJ, Stubbs NC (2011). Swing phase kinematic and kinetic response to weighting the hind pasterns. Equine Vet J.

[37] Colborne GR (2008). Are sound dogs mechanically symmetric at trot? No, actually. Vet Comp Orthop Traumatol.

[38] Poy NSJ, DeCamp CE, Bennett RL, Hauptman JG (2000). Additional kinematic variables to describe differences in the trot between clinically normal dogs and dogs with hip dysplasia. Am J Vet Res.

[39] Owen MR, Richards J, Clements DN, Drew ST, Bennett D, Carmichael S (2004). Kinematics of the elbow and stifle joints in greyhounds during treadmill trotting—an investigation of familiarisation. Vet Comp Orthop Traumatol.

[40] Herbin M, Hackert R, Gasc JP, Renous S (2007). Gait parameters of treadmill versus overground locomotion in mouse. Behav Brain Res.

